# Rating general practitioner consultation performance in cancer care: does the specialty of assessors matter? A simulated patient study

**DOI:** 10.1186/1471-2296-15-152

**Published:** 2014-09-13

**Authors:** Moyez Jiwa, Georgia Halkett, Xingqiong Meng, Melissa Berg

**Affiliations:** Curtin University, GPO Box U1987, Perth, Australia

**Keywords:** Radiation therapy, Prostate cancer, General practitioner, Consultation, Assessment, Side effects

## Abstract

**Background:**

Patients treated for prostate cancer may present to general practitioners (GPs) for treatment follow up, but may be reticent to have their consultations recorded. Therefore the use of simulated patients allows practitioner consultations to be rated. The aim of this study was to determine whether the speciality of the assessor has an impact on how GP consultation performance is rated.

**Methods:**

Six pairs of scenarios were developed for professional actors in two series of consultations by GPs. The scenarios included: chronic radiation proctitis, Prostate Specific Antigen (PSA) ‘bounce’, recurrence of cancer, urethral stricture, erectile dysfunction and depression or anxiety. Participating GPs were furnished with the patient’s past medical history, current medication, prostate cancer details and treatment, details of physical examinations. Consultations were video recorded and assessed for quality by two sets of assessors- a team of two GPs and two Radiation Oncologists deploying the Leicester Assessment Package (LAP). LAP scores by the GPs and Radiation Oncologists were compared.

**Results:**

Eight GPs participated. In Series 1 the range of LAP scores by GP assessors was 61%-80%, and 67%-86% for Radiation Oncologist assessors. The range for GP LAP scores in Series 2 was 51%- 82%, and 56%-89% for Radiation Oncologist assessors. Within GP assessor correlations for LAP scores were 0.31 and 0.87 in Series 1 and 2 respectively. Within Radiation Oncologist assessor correlations were 0.50 and 0.72 in Series 1 and 2 respectively. Radiation Oncologist and GP assessor scores were significantly different for 4 doctors and for some scenarios. Anticipatory care was the only domain where GPs scored participants higher than Radiation Oncologist assessors.

**Conclusion:**

The assessment of GP consultation performance is not consistent across assessors from different disciplines even when they deploy the same assessment tool.

## Background

It is important that the general practitioners (GPs) who are consulted by patients treated for prostate cancer are able to consult skilfully. Patients with prostate cancer often experience stigma by virtue of the psychosocial impact of the disease [1]. Many such patients have unmet needs which may not be explicitly presented and yet expect to be supported by their GP [2–4]. Relative to some conditions (e.g. diabetes) patients who have completed treatment for cancer present infrequently in general practice [5]. Consultations with ‘real’ patients are challenging to record and analyse. It is difficult to reliably report GP performance at individual consultations for appropriate diagnosis and management of cancer related issues given the myriad of possible confounding factors, including the need to recruit ill or distressed patients [6]. Also comparison is only possible when the patient presents consistently to all GP participants. Therefore, the deployment of actor-patient simulations (henceforth called simulated patients) in which confounding variables can be controlled is a possible solution to explore GP cancer care [7]. The use of ‘live’ simulations in which it is possible to observe the interaction between ‘patient’ and doctor adds to the validity of the data. Research deploying simulated patients have been reported in the literature [8–10]. A number of limitations exist with the use of simulations, including the validity of the assessment scores [11]. This paper will focuses on the assessment of GP performance in such consultations. In previous research assessors from different specialities have used different assessment measures and have noted significant differences in GP assessment and management of patients. This is difficult to interpret [9]. A particular criticism of this approach is that doctor consultation performance is being measured on different benchmarks, by assessors with different backgrounds. Comparisons may be neither reliable nor valid. This is especially important when reviewing the care of people who have been treated for cancer. In the present study GP consultation performance is rated on the same measure, but by assessors from different specialties. The aim of this study was to determine whether the speciality of the assessor affects how GP consultation performance is rated.

## Methods

This study was approved by the Human Research Ethics Committee at Curtin University (RD-12-10).

Simulated consultations were video-recorded and assessed by two GP assessors and two Radiation Oncologist assessors.

### Scenarios

Six pairs of scenarios were developed for two series of consultations by the research team using previous literature and with reference to two radiation oncologists, two GPs, a cancer nurse coordinator and a urologist. The scenarios included: chronic radiation proctitis, PSA bounce, recurrence of cancer, urethral stricture, erectile dysfunction and depression or anxiety.

#### Series 1 scenarios

**Diagnosis**

Chronic radiation proctitis.

Prostate Specific Antigen (PSA) bounce after radiation therapy.

Recurrence of prostate cancer with bone metastases.

Urethral stricture after radiotherapy. Late urinary toxicity. Not infection.

Erectile dysfunction after radiation therapy.

Depression after prostate cancer treatment.

#### Series 2 scenarios

**Diagnosis**

Late radiation bowel toxicity: Radiation proctitis OR Radiation proctopathy.

PSA elevation post radiotherapy.

Recurrence of prostate cancer with spinal bone metastases.

Urinary symptoms post-Brachytherapy. No infection.

Erectile Dysfunction (ED), post-treatment for prostate cancer.

Anxiety after prostate cancer treatment.

These scenarios were selected as those that might be presented to a GP for advice [12, 13]. They were performed by professional actors. Information available to the GPs at the time of the consultation included the patient’s past medical history, current medication, prostate cancer details and treatment, details of physical examinations.

### GP participants

Participants were recruited by convenience sampling through personal contact with the research team.

Series 1: Six GPs participated in Series 1 (males = 4, females = 2). Participants were offered brief feedback on the correct diagnosis and management of each case.

Series 2: Five GPs participated in Series 2 (males = 2, females = 3) including 3 GPs who participated in Series 1.

### Consultations

GPs were invited to consult with the simulated patients as though the person had previously visited the practice for ongoing medical problems. The GPs were aware that the ‘patient’ presenting to their clinic was an actor. A medical record with the relevant past medical history was prepared for each patient and was made available to the GP. Physical examination cards, describing examination findings were presented to the GP by the simulated patient when requested by the doctor. No ‘patient’ was actually examined. The practitioners were allowed up to 15 minutes and the consultation was video recorded. The scenarios were presented to the GP participants as a series of consecutive cases. Scenarios were presented in a random order to GPs in each series.

### Quality of consultation

The Leicester Assessment Package (LAP) is an established measure of consultation competence for general practice consultations [14]. Five of the seven LAP categories of consultation competence (interviewing and history taking, problem solving, patient management, anticipatory care and behaviour/relationship with patients) were assessed in this study.

### Assessment by GPs and Radiation Oncologists

The recordings were independently rated by two GPs, who had previously assessed consultations using LAP scoring [9, 15]. The GP assessors then compared scores and agreed on a consensus score which represented the quality of the consultations. Two Radiation Oncologists were also trained by a GP familiar with LAP scoring. The Radiation Oncologists assessed the consultations independently and then compared their LAP scores to derive a consensus score. Similar scoring was completed for both series of consultations by all the assessors. Each participating GP was given a total score for each scenario, one from the GP LAP assessors and one from the Radiation Oncology LAP assessors.

### Sample size

Power calculations for correlated data using simulations, to avoid an underlying assumption of normality, indicated that a sample of 5 doctors with each doctor completing 6 scenarios would be sufficient to detect an intraclass correlation coefficient (ICC) of 0.75 in LAP scores between doctors at 90% power and 5% level of significance, assuming null ICC is 0.10. Sample size calculations were performed using the ‘sampicc’ command in Stata®.

### Statistical analysis

The mean LAP scores from both the GP and Radiation Oncologist assessors for each GP participant were calculated. The differences in mean scores between individual doctors were then estimated in a standard unadjusted linear regression model. In order to determine whether the mean LAP scores varied by each of the simulated patient scenarios, Generalised Estimating Equations (GEEs) were used to fit a linear model using simulated patient scenario as the single independent variable (Table 1). The mean differences between GP and Radiation Oncologist assessors relating to GP performance in each clinical domain (e.g. “interviewing/history taking”, etc.) were evaluated by using the multilevel mixed effect models, with patients nested in GPs and GPs nested in assessors (Table 2). All analyses were performed using Stata (Intercooled 9.2, StataCorp, College Station, TX, USA).

**Table 1:** **Comparison of GP and Radiation Oncologist assessors scores; mean of consultations for each participant (doctor)**

	GP Assessor mean LAP score				Radiation Oncologist mean LAP score				Mean difference	p value
Doctor	Mean	SD	β	p-value	Mean	SD	β	p-value	Radiation Oncologist – GP	
Series 1										
1	70.0	3.7	0.0	-	81.4	3.0	0.0		11.5	.001
2	75.5	3.2	5.5	.03	80.9	2.7	−0.5	.81	5.5	.01
3	76.6	2.2	6.6	.01	74.5	5.3	−6.9	.001	−2.1	.39
4	68.6	4.9	−1.4	.58	72.1	1.1	−9.3	<.001	3.5	.12
5	70.7	6.6	0.8	.75	76.6	2.6	−4.8	.02	5.8	.07
6	74.6	3.3	4.6	.07	78.4	4.0	−3.0	.14	3.9	.10
Series 2										
1	77.1	3.8	0.0	-	82.5	5.8	0.0	-	5.4	.09
2	77.5	4.3	0.4	.86	82.0	3.3	−0.4	.87	4.6	.07
3	63.1	4.4	−14.0	<.001	77.6	2.9	−4.9	.07	14.5	<.001
7	77.5	4.9	0.4	.86	73.8	2.7	−8.7	.002	−3.7	.14
8	54.4	2.3	−22.7	<.001	64.5	6.1	−18.0	<.001	10.1	.004

β: mean difference in scores (coefficient) relative to doctor number 1.

**Table 2:** **Comparison of assessor scores for each LAP domain in both series; mean of all consultations**

	GP assessors		Rad Onc assessors		Mean difference (Rad Onc – GP)		
	Mean	S.D.	Mean	S.D.	β coefficient	Standard error	P value
Series 1							
Interviewing/History taking	72.7	5.5	78.1	4.5	5.3	1.02	<.001
Patient management	71.4	5.1	78.9	4.6	7.6	1.05	<.001
Problem solving	73.7	6.8	76.0	8.6	2.3	1.48	0.120
Behaviour/relationship with patients	72.4	7.5	80.6	7.4	8.1	1.57	<.001
Anticipatory care	73.8	11.9	68.7	9.9	−5.1	4.14	0.220
Series 2							
Interviewing/History taking	71.5	10.0	77.0	7.6	5.5	1.10	<.001
Patient management	68.3	10.7	74.6	10.5	6.4	1.71	<.001
Problem solving	69.3	11.9	75.8	8.3	6.4	1.66	<.001
Behaviour/relationship with patients	71.2	13.4	78.1	10.7	6.9	2.01	0.001
Anticipatory care	80.0	0.0	74.3	9.6	−5.7	9.04	0.530

p values were derived from multilevel mixed effect models.

## Results

### Comparison between scores from GP and Radiation Oncology assessors using LAP scoring

LAP scores by the GPs and Radiation Oncologist assessors were collated and compared. The overall range for GP LAP scores in Series 1 was 61%-80% and the overall range for Radiation Oncologist LAP scores in Series 1 was 67%-86%. The overall range for GP LAP scores in Series 2 was 51%-82% and the overall range for Radiation Oncologist LAP scores in Series 2 was 56%-89%. These LAP scores are consistent with scores achieved in our previous work [9, 15].

LAP scores for each series were then compared using linear regression models to determine whether GP and Radiation Oncologist assessors similarly scored participating GPs (Table 1). The mean difference in scores (β coefficient) relative to doctor number 1 and associated p-value are also shown as estimated from each of the four standard unadjusted linear regression models. Radiation Oncologist and GP assessor scores were significantly different on 4 occasions. Radiation Oncologist assessors mean scores per consultation were higher than the GP assessors mean scores.

Within GP assessor correlations (Intraclass Correlation Coefficients) for LAP scores were 0.31 and 0.87 in Series 1 and 2 respectively. Within Radiation Oncologist assessor correlations were 0.50 and 0.72 in Series 1 and 2 respectively.

### LAP scoring by domains

Because we detected a difference in the scoring between GP and Radiation Oncologist assessment we explored whether the scores varied depending on the different domains within the LAP. Table 2 provides a comparison of GP and Radiation Oncologist assessor scores by LAP domain. Radiation Oncologist assessors LAP scores were significantly higher for most domains in both series, apart from problem solving in series one and anticipatory care in both series where the scores were not significantly different.

## Discussion

In a previous study with simulated patients the type of assessment for consultation performance as well as the background of the assessors was reported to be relevant [9]. In this study Radiation Oncologists assessors rated GP consultation better than GP assessors on almost every domain of the same tool. Relatively underperforming GPs were scored significantly better by specialists than by GPs.

The use of simulated patients has long been established to assess practitioner performance [16]. A recent review concludes that

*“..correlations with written examinations were modest, adding empirical support to the notion that standardized patients assess aspects of competence not addressed directly by the traditional written measures.”* [17]

However, the issue of the expertise and background of the assessor of simulated patient consultations receives limited attention in reviews around assessment of practitioners [18]. For example, in medical training courses practitioners are mentored and assessed by a variety of practitioners and yet may choose a career in general practice [19]. This assessment could be improved by ensuring that the consultation skills are assessed by a person practicing in the target specialty [19]. Anticipatory care was the only domain of LAP where GPs appeared to score higher than radiation oncologists, this may reflect the value of ensuring continuity of care in general practice [20].

The strength of this study was the use of simulated patients to research assessment of consultation skills in the management of defined issues presented by a specific group of interest. There are three key limitations; firstly the technique offers a proxy measure for clinical competence and the validity of this method may be diminished by any undetected differences in simulated patient performance. However, experts agree that simulated patient studies are valid in assessing consultation skills [11]. Also the patients were not physically examined and the value of skilful examination in facilitating disclosure of symptoms may be significant. Secondly, the training of the assessors may have had a bearing on the scores assigned. It is also possible that the two specialist practitioners were atypically generous in their assessment. It has long been recognised that assessors in the same specialty also vary [11]. Finally, the practitioners in this study all performed well with relatively high LAP scores compared to performance rated in other simulated patient studies [8, 9].

## Conclusion

This is the second study using simulated patients in which we report that the management of patients with problems related to or associated with prostate cancer treatment is challenging [21]. The assessment of GP consultation performance in this context is not consistent across assessors from different disciplines even when they deploy the same assessment tool. Future research might explore the question of whether LAP scores based on consultations where management is inappropriate also vary. In other words, do specialists and GPs differ in their assessment and recognition of errors in practice?

## Abbreviations

GPs: General practitioners
PSA: Prostate Specific Antigen
LAP: Leicester Assessment Package.
